# Cancer Stemness Associated With Prognosis and the Efficacy of Immunotherapy in Adrenocortical Carcinoma

**DOI:** 10.3389/fonc.2021.651622

**Published:** 2021-07-21

**Authors:** Xiaoxi Shi, Yuanlin Liu, Shuai Cheng, Haidi Hu, Jian Zhang, Minjie Wei, Lin Zhao, Shijie Xin

**Affiliations:** ^1^ Department of Vascular and Thyroid Surgery, The First Hospital, China Medical University, Shenyang, China; ^2^ Department of Pharmacology, School of Pharmacy, China Medical University, Shenyang, China

**Keywords:** adrenocortical carcinoma, cancer stemness, immune cell infiltration, immune checkpoint inhibitors, WGCNA

## Abstract

**Background:**

Cancer stem cells (CSCs) have been proven to influence drug resistance, recurrence, and metastasis in tumors. Our study aimed to identify stemness-related prognostic biomarkers for new therapeutic strategies in adrenocortical carcinoma.

**Methods:**

RNA-seq data and clinical characteristics were downloaded from The Cancer Genome Atlas (TCGA). The stemness indexes, mDNAsi and mRNAsi, were calculated to classify all samples into low-score and high-score groups. Two algorithms, based on the R language, ESTIMATE and single-sample Gene Set Enrichment Analysis (ssGSEA) were used to assess the immune cell infiltration states of adrenocortical carcinoma patients. Weighted Gene Co-expression Network Analysis (WGCNA) was used to find genes that were related to the stemness of cancer. By bioinformatics methods, the correlations between biomarkers capable of predicting immune checkpoint inhibitors (ICIs) responses and stemness of cancer were explored.

**Results:**

High-mRNAsi predicted shorter overall survival (OS) and a higher metastatic trend in adrenocortical carcinoma (ACC) patients. Compared with the low-mRNAsi group, the high-mRNAsi group had a lower ImmuneScore and StromalScroe. Twenty-two stemness-related prognostic genes were obtained by WGCNA, which focused on the function of the cell cycle and cell mitosis. Immune cell infiltration, especially CD8+T cell, increased in the low-mRNAsi group compared with the high-mRNAsi group. Lower expression of PD-L1, CTLA-4, and TIGHT was evaluated in the high-mRNAsi group.

**Conclusions:**

ACC patients with high-mRNAsi have poor prognosis and less immune cell infiltration. Combined with the finding of lower expression of CTLA-4, TIGHT, and PD-L1 in the high-mRNAsi group, we came to the conclusion that stemness index is a potential biomarker to predict the effectiveness of ICIs.

## Introduction

Adrenocortical carcinoma (ACC) is a rare malignancy with reported incidence of 0.7 to 2 cases per million per year (1). The age at diagnosis follows a bimodal pattern of age distribution, mainly before the age of five and between the fourth and fifth decades of life (2). And Cushing’s syndrome is observed in 50–60% of patients with ACC (3). Hyperandrogenism is seen in 20–30% of female patients, with a small number of those patients having estrogen and/or mineralocorticoid excess. Primary aldosteronism can also be seen in only 2.5% of patients with ACC (4). Although complete tumor resection accompanied with adjuvant mitotane therapy affords an opportunity to cure patients, the prognosis of adrenocortical carcinoma patients is generally poor with a median OS of around 15 months (5). Now, target immunotherapy has been successfully used in many kinds of cancers, such as breast cancer (6) and ovarian cancer (7), so it is necessary to make breakthroughs in ACC treatment.

Now, research of cancer stem cells, which are defined as cells that have the characteristics of stem cells, the capacity of self-renewal, and can promote tumor cells to invade and grow in cancer, has shown the unique function of cancer stem cells (8). They have the potential to generate all cell types. Furthermore, cancer stem cells seem to be less susceptible to chemotherapy due to their unique characteristics. And many studies have also indicated that cancer stem cells play a vital role in the process of metastasis and differentiation of cancer (9, 10). Therefore, to improve the therapeutic efficacy of ACC, it is necessary to deeply understand cancer stem cells.

In order to better understand and describe the unique characteristics of cancer stem cells, by deep learning methods, Malta et al. established a scoring system, using the One-Class Logistic Regression (OCLR) machine learning algorithm to compare the similarity between tumor cells and different types of stem cells, which were obtained from the Progenitor Cell Biology Consortium (https://www.synapse.org/pcbc), and thus obtained two stemness indexes, mDNAsi and mRNAsi (11). The mDNAsi index was based on the DNA methylation level and reflected the epigenetic stemness features, while the mRNAsi index was based on mRNA expression level and reflected the transcriptomic stemness features. We found that cancer stemness was correlated with the biomarkers to predict response to immune checkpoint inhibitors, which may reveal possible potential therapeutic targets.

The immune checkpoint inhibitor has changed the clinical therapeutic strategy, which provides an alternative effective therapeutic option for ACC patients (12). But the efficacy and application conditions of immune checkpoint inhibitors in advanced ACC patients is still unclear (13). Our study comprehensively analyzed the stemness of adrenal tumors especially ACC in a TCGA cohort (n = 232). First, we analyzed the correlations between stemness index and prognosis and clinical characteristics of ACC. Second, by WGCNA and function analysis, the biological functions of stemness-related genes were explored. Further more, the correlations between stemness index and ICIs-associated biomarkers such as immune cell infiltration, tumor mutation burden (TMB), and mismatch repair (MMR)-related gene expression were systematically analyzed, which would provide a reference for future diagnosis and therapy of ACC. The results indicated that the ACC patients with different level of stemness indexes or stemness-related gene expression may have different efficacy of immune checkpoint inhibitors.

## Materials and Methods

### Materials

In our study, we downloaded the clinical characteristics and RNA-seq data of 232 adrenal tumor samples (148 PCPG and 78 ACC) from the TCGA database (https://portal.gdc.cancer.gov/).

And the stemness indexes (mRNAsi and mDNAsi) were obtained from previous research (11), which was based on a One-Class Logistic Regression (OCLR) machine learning algorithm (14). Stemness indexes reflected the similarity between tumor cells and stem cells, mDNAsi reflected the epigenetic stemness features and mRNAsi reflected the transcriptomic stemness features.

### Prognosis and Clinical Characteristics Analysis of Stemness Index in Adrenal Tumor

The stemness indexes (mRNAsi and mDNAsi), which are continuous variables, were divided into low-score and high-score groups by the median value. We used Kaplan-Meier (K-M) analysis and log-rank tests to compare the difference between the two groups. We extracted the clinical characteristics (age, gender, histological type, T stage, N stage, M stage, tumor stage) to explore the association between them and the stemness indexes. We used the Wilcoxon rank sum test for age, gender, N stage, and M stage, and Dunn’s test for histological type and T stage. All statistical analyses were completed in the R language (Version 4.0.2), and visualized by R package “ggplot2”. The results are shown in **Table 1 **in the** Supplementary Material**.

### Tumor Microenvironment and Immune Cell Infiltration Analysis of Stemness Index in Adrenal Tumor

#### Estimate

We used the R package “ESTIMATE” to evaluate the tumor microenvironment (TME) for each adrenal tumor sample (15), which included ESTIMATE score (stromal-immune comprehensive score), immune score (immune cell infiltration), tumor purity, and stromal score (stromal content).

#### Single-Sample Gene-Set Enrichment Analysis

ssGSEA was preformed to check 28 kinds of immune cell infiltration states in each ACC sample (16). In order to compare the data, x was replaced with x with the formula x= (x-min(x))/(max(x)-min(x)) for each ssGSEA score, where min(x) and max(x) represented the minimum and maximum values of the ssGSEA score in the ACC sample.

### WGCNA Reveals the Stemness-Related Hub Genes

The “DESeq2” package of the R language was used to identify differentially expressed genes (DEGs) between high-miRNAsi and low-miRNAsi groups (|log2FC| > 1 and P < 0.05).

WGCNA was performed with the R package “WGCNA” (17). DEGs in the same module meant they had correlations in expression. We chose 0.25 as the threshold to merge modules with similar expression. And then we chose the module membership (MM) > 0.8 and gene significance (GS) > 0.5 to identify the hub genes.

### GO and KEGG Analyses

The biological functions of stemness-related hub genes was explored and determined by GO and KEGG analyses, which were performed by R package “clusterProfiler”.

### Protein-Protein Interaction (PPI) Network Analysis

A PPI network was established in the STRING database (https://www.string-db.org) to analyze the correlation of stemness-related hub genes at the protein level.

### Calculation of Tumor Mutation Burden (TMB)

Nucleotide variation data downloaded from TCGA was used to calculate the TMB by the R package “maftools” (18).

### Prediction of the Half-Maximal Inhibitory Concentration (IC50)

The effect of chemotherapy was predicted by R package “pRRophetic” (19), which was based on a ridge regression model to calculate the half-maximal inhibitory concentration (IC50) of drugs.

### Statistical Analyses

All statistical analyses were completed in the R language (Version 4.0.2). Correlation analyses were performed and visualized by R package “corrplot”. We used the Wilcox test and t-test to calculate the difference between high-mRNAsi and low-mRNAsi groups. Univariate Cox proportional hazards regression was used to analyze the independent hazard ratio of the stemness-related hub genes. The log-rank method was used to calculate significant survival P-values. P < 0.05 was considered statistically significant.

## Results

### Stemness Index Correlated With the Prognosis and Clinical Characteristics of Adrenocortical Carcinoma

**Figure 1:**
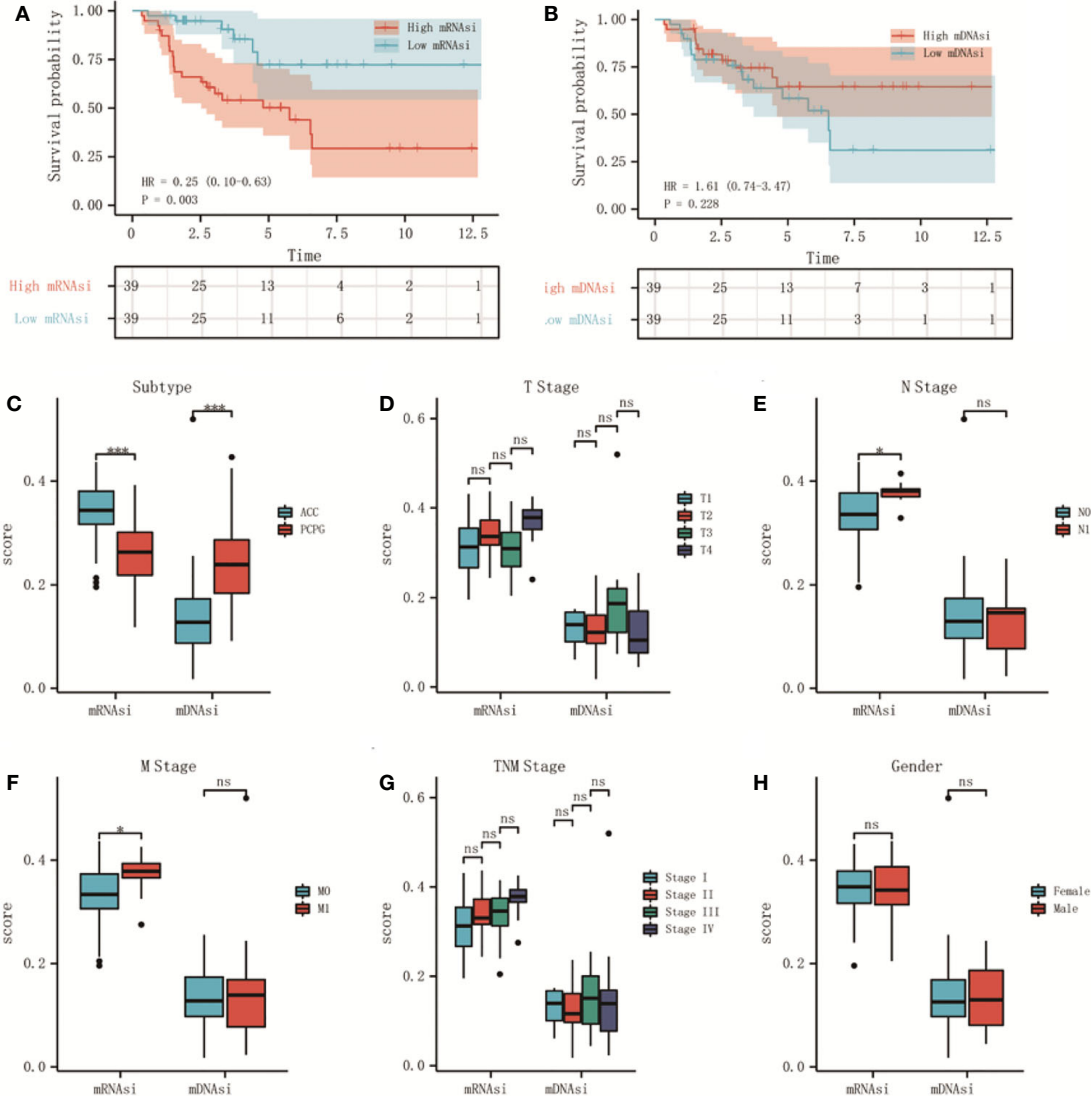
The relationship between stemness indexes (mRNAsi and mDNAsi) and prognosis and clinical characteristics in adrenal tumors. **(A, B)** Kaplan–Meier OS curves for ACC **(A)** and PCPG **(B)** patients with low and high mRNAsi based on the median cut-off point. **(C, D)** Kaplan–Meier OS curves for ACC **(C)** and PCPG **(D)** patients with low and high mDNAsi based on the median cut-off point. **(E)** Boxplot of mRNAsi and mDNAsi in individual samples of ACC and PCPG patients. **(F–J)** Boxplots of mRNAsi and mDNAsi in individual samples of ACC stratified by T stage **(F)**, N stage **(G)**, M stage **(H)**, tumor stage **(I)**, and gender **(J)**. ***P < 0.001; ns, not significant).

We classified adrenal tumors patients into high-score and low-score groups by the median value of mRNAsi or mDNAsi in ACC. In ACC patients, the results of Kaplan-Meier survival analysis suggested that the patients in the high-mRNAsi group had shorter OS than those in the low-mRNAsi group (P < 0.01) (**Figure 1A**), and no significant difference was found between high-mDNAsi and low-mDNAsi groups (P = 0.228) (**Figure 1B**). So, we further performed correlation analyses of clinical characteristics with stemness indexes in ACC patients.

As shown, there was a significant difference in different histological types of adrenal tumor, mRNAsi was higher in ACC but lower in pheochromocytoma and paraganglioma (PCPG) cancers (**Figure 1C**). Moreover, 78 ACC patients were classified by clinical characteristics, which were downloaded from the TCGA database, including T stage, N stage, M stage, tumor stage, and gender. For N stage, mRNAsi showed a higher trend N1 stage (**Figure 1E**). Similarly, mRNAsi was significantly related to metastatic status (**Figure 1F**). However, for mDNAsi, it was not associated with any clinical characteristics (**Figures 1D, G, H**). The information about clinical features is shown in **Table 2 **in the** Supplementary Material**.

### Stemness Index Correlated With the Tumor Microenvironment of Adrenocortical Carcinoma

**Figure 2:**
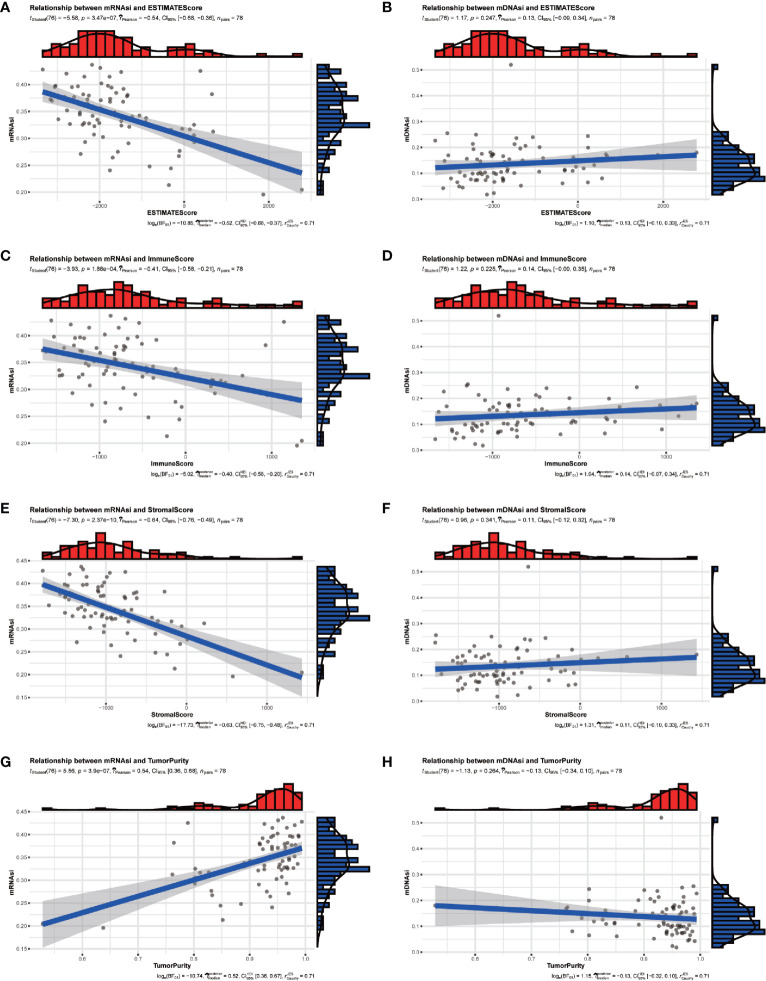
The relationship between stemness indexes (mRNAsi and mDNAsi) and immune microenvironment in ACC patients. **(A, B)** Correlation between ESTIMATEScore and mRNAsi **(A)**, mDNAsi **(B)**. **(C, D)** Correlation between ImmuneScore and mRNAsi **(C)**, mDNAsi **(D)**. **(E, F)** Correlation between StromalScore and mRNAsi **(E)**, mDNAsi **(F)**. **(G, H)** Correlation between TumorPurity and mRNAsi **(G)**, mDNAsi **(H)**.

The tumor microenvironment of ACC patients was analyzed by R package “ESTIMATE”. The results indicated that mRNAsi had a significant positive correlation with tumor purity (**Figure 2G**) and negative correlation with ESTIMATE scores (**Figure 2A**), immune scores (**Figure 2C**), and stromal scores (**Figure 2E**). But there was no significant correlation between mDNAsi and those scores (**Figures 2B, D, F, H**).

In summary, our grouping of stemness had an excellent discrimination ability for clinical characteristics and the tumor microenvironment.

### Identification of the Stemness-Related Module and Genes in ACC Patients

**Figure 3:**
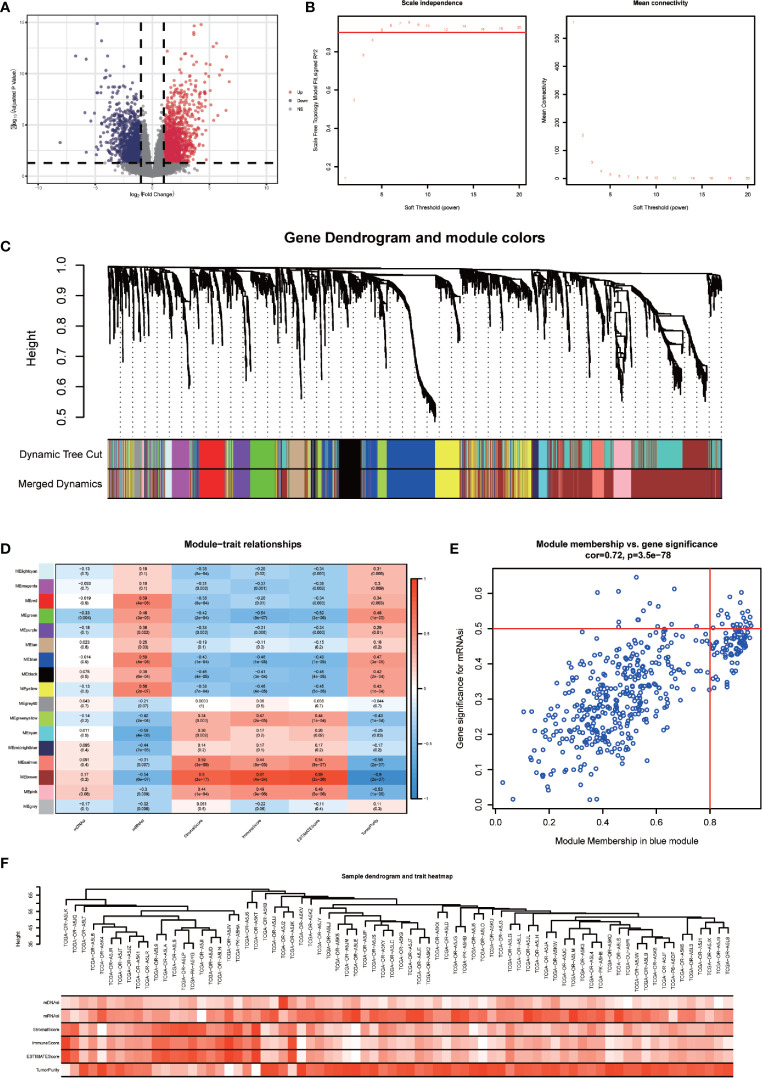
Identification of stemness-related hub genes through WGCNA. **(A)** Volcano plots of DEGs between high-mRNAsi and low-mRNAsi groups. **(B)** Analysis of the scale-free fit index for various soft-thresholding powers (β) and the mean connectivity for various soft-thresholding powers. **(C)** TOM cluster dendrogram of WGCNA: Branches with different colors corresponding to different modules. Dynamic Tree Cut represents the original module, while Merged Dynamic represents the final module. **(D)** Correlation of traits (mDNAsi, mRNAsi, ESTIMATEScore, StromalScore, ImmuneScore, and TumorPurity) with modules. Colors representing a gene set, the correlation coefficient, and P-value have been marked. **(E)** Scatter plot of module eigengenes in the blue module with cut-offs of MM > 0.8 and GS > 0.5. **(F)** Heatmap of the clustering of 78 ACC patients.

DEGs between the high-mRNAsi and low-mRNAsi groups were identified through the R package “DESeq2”. A total of 3210 DEGs were obtained, of which 1788 were upregulated and 1422 were downregulated (P < 0.05 and |log2FC| > 1). The volcano map was drawn (**Figure 3A**). Based on the DEGs, a co-expression network was established by R package “WGCNA”, which could reveal the modules and genes that were significantly associated with the tumor microenvironment and mRNAsi. In this study, β = 4 was the best choice for soft thresholds to construct a scale-free network (**Figure 3B**). After adjusting the parameters of WGCNA, we classified the DEGs into 17 modules (**Figure 3C**). The heatmap showed that the blue module was both correlated with mRNAsi (p < 0.001) and TME (p < 0.001) (**Figure 3D**). Afterward, we defined 22 genes in the blue module with GS > 0.5 and MM > 0.8 as stemness-related hub genes (**Figure 3E**), including CDCA8, STIL, CCNB1, CDC25C, MTFR2, PSRC1, CDCA5, CDKN3, DLGAP5, ARHGAP11A, BUB1B, CASC5, CCNB2, KIF23, UBE2T, TYMS, AURKA, RRM2, APOBEC3B, CENPA, KIF4A, and SPC25. The clustering result of 78 ACC patients was shown in the heatmap (**Figure 3F**).

### Functional and Correlation Analysis of Hub Genes

**Figure 4:**
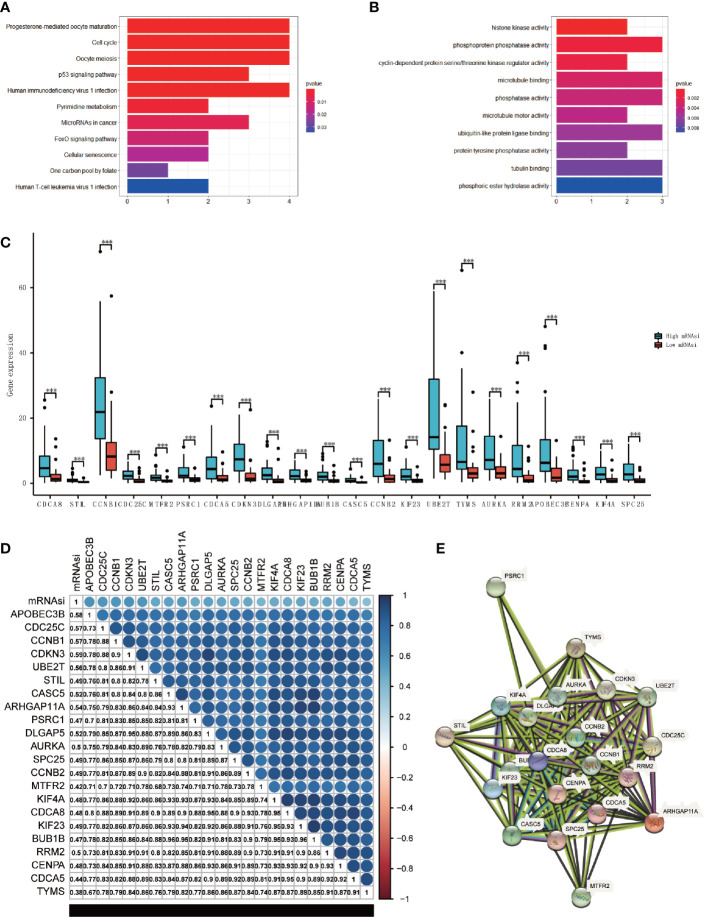
Analysis of the main function and the correlation of hub genes. **(A, B)** KEGG **(A)** and GO **(B)** analysis of the hub genes. **(C)** Boxplot of the differential analysis of the expression of 22 hub genes between high-mRNAsi and low-mRNAsi groups (***P < 0.001). **(D)** Correlation analysis between 22 hub genes, correlation coefficient have been shown (P < 0.05). **(E)** The protein-protein interactions between the hub genes.

We performed GO and KEGG enrichment analyses to further evaluate the functions and pathway of the hub genes. The results of KEGG analysis indicated that the major pathways of hub genes were oocyte meiosis (hsa04114) and cell cycle (hsa04110) (**Figure 4A**). The GO enrichment analysis was composed of three portions: biological process (BP), cell component (CC), and molecular function (MF), indicating that the major biological function of the hub genes were cyclin-dependent protein serine/threonine kinase regulator activity (GO:0016538), microtubule binding (GO:0008017), and phosphatase activity (GO:0016791) (**Figure 4B**). These analyses proved that hub genes were significantly related to cell cycle events and immune reaction.

We came to the conclusion that all of the hub genes were overexpressed in the high-mRNAsi group through analyzing the expression values of each hub gene (**Figure 4C**). The correlation analyses were also performed to check the expression correlation between the hub genes. Surprisingly, results showed a high positive correlation among all hub genes (P<0.05), and these hub genes were positively correlated with mRNAsi (**Figure 4D**). Additionally, at the protein level, we constructed a PPI network using STRING (**Figure 4E**), which consisted of 22 nodes and 172 edges, and 4 hub genes (CCNB1, CCNB2, CDCA8, CENPA) played key roles. Only APOBEC3B had no interaction with the others. The analysis of two validation sets (GSE90713 and GSE143383) was preformed to validate the findings, the results also showed that the hub genes were higher expressed in the ACC patients compared with normal people (**Figure S3** in the **Supplementary Material**). The protein expression level of hub genes was verified by The Human Protein Atlas (**Figure S4 **in the** Supplementary Material**).

### Stemness‐Related Hub Genes Associated With Prognosis and Cytotoxic Chemotherapies

**Figure 5:**
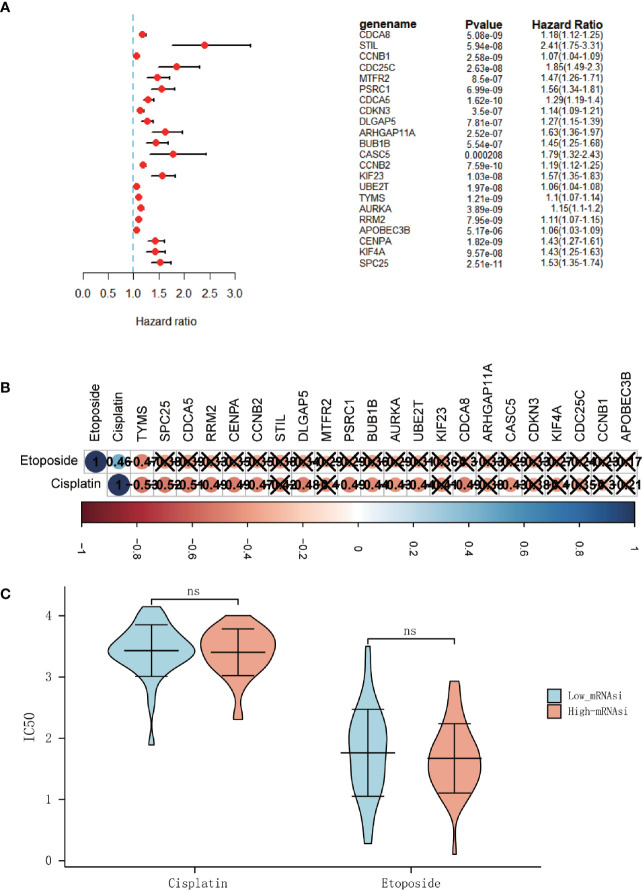
The hub genes associated with overall survival (OS) in ACC patients. **(A)** Univariate Cox regression analysis between hub genes and OS in ACC patients. **(B)** Correlation analysis between 22 hub genes and IC50 of cisplatin and etoposide (**×** means P > 0.05). **(C)** Differential analysis of the IC50 of the cisplatin and etoposide between high-mRNAsi and low-mRNAsi groups. (ns, not significant).

The results of univariate analyses for each hub gene showed that 22 genes were both risk factors for OS in ACC patients (p<0.05) (**Figure 5A**). Kaplan-Meier survival analysis suggested the same results (P < 0.01, **Figure S1**, **Figure S2**). The above analyses proved that stemness‐related hub genes and mRNAsi were significantly related to prognosis in ACC patients. It indicated that stemness‐related hub genes and mRNAsi must have unique and critical action in the tumor progression, which might be a target to formulate clinical treatment strategies. Multivariate cox analysis of mRNAsi and other clinical characteristics showed that except for T stage, no other characteristics was relevant to the prognosis (P<0.05) (**Table 3 **in the** Supplementary Material**. It may be due to the relatively small sample size.

And since platinum-based chemotherapy combined with etoposide and doxorubicin plus mitotane (EDP-M scheme) is the first line of treatment in advanced ACC (20), the R package “pRRophetic” was used to predict the IC50 of chemotherapy drugs that are commonly used in ACC treatment, including cisplatin and etoposide. But it was disappointing that no significantly difference of the half-maximal inhibitory concentration (IC50) was found between the high-mRNAsi and low-miRNAsi groups (**Figure 5C**). And only a few genes were found to be related to the IC50 of typical chemotherapy drugs (**Figure 5B**).

### Immune Cell Infiltration Decreased With the Increase of mRNAsi Index

**Figure 6:**
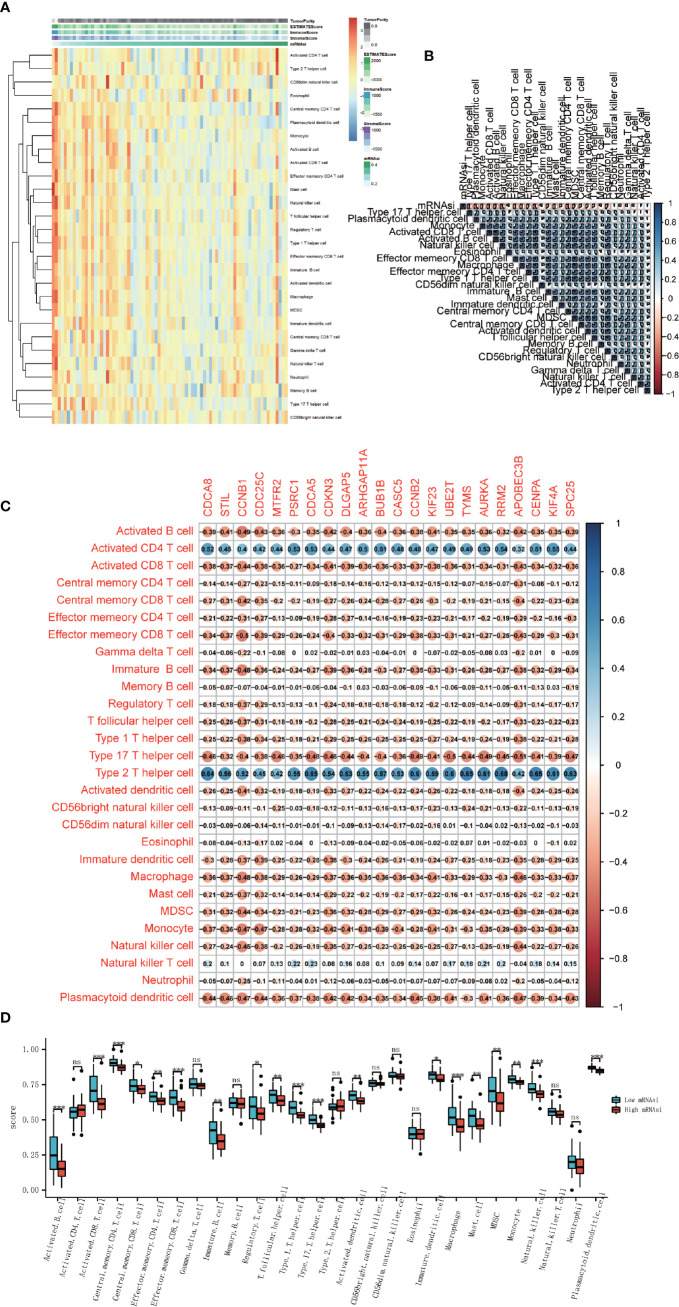
Association between immune cell infiltration and cancer stemness. **(A)** Heatmap of the ssGSEA score calculated by immune cell gene sets. **(B)** Correlation analysis between 28 immune cells and mRNAsi score. **(C)** Correlation analysis between 28 immune cells and 22 stemness-related hub genes. **(D)** Boxplot of the differential analysis of 28 immune cells’ infiltration between high-mRNAsi and low-mRNAsi groups (*P < 0.05, **P < 0.01, ***P < 0.001, ns, not significant).

We performed ssGSEA to evaluate the abundance of 28 immune cells in all ACC patients. Results showed that with the increase of mRNAsi, immune cells infiltration decreased (**Figure 6A**). Correlation analysis of immune cell infiltration based on the results of ssGSEA was also carried out and showed a high positive correlation among all immune cells, and these immune cells were negatively correlated with the mRNAsi index (**Figure 6B**). The different abundance of immune cells between high-mRNAsi and low-mRNAsi groups is shown in the boxplot (**Figure 6D**). Except for activated CD4 T cells, gamma delta T cells, memory B cells, type 2 T helper cells, CD56 bright natural killer cells, CD56 dim natural killer cells, eosinophils, natural killer T cells, and neutrophils, other immune cells were downregulated significantly in the high-mRNAsi group. The expression of stemness‐related hub genes were correlated with some kinds of immune cells, including activated B cells, activated CD4 T cells, activated CD8 T cells, type 2 T helper cells, and type 17 T helper cells, while activated CD4 T cells and type 2 T helper cells were positively correlated (**Figure 6C**). It is worth noting that in previous research, ICIs were believed to work in large part by awakening pre-existing tumor immune responses. A tumor with lower infiltration of CD8 + T cells showed more resistance to immunotherapy, thus, tumors with low infiltration of CD8 + T cells generally had worse clinical outcomes (21). So, in combination with our above analysis, it is reasonable to consider that mRNAsi and stemness‐related hub genes are associated with the effect of the immunotherapy.

### Stemness Index Related to the Biomarkers of Immune Checkpoint Inhibitors

Since immune checkpoint inhibitors may constitute an effective therapeutic strategy, next, we investigated some biomarkers that could predict the response to immune checkpoint inhibitors which have been proven. Some potential predictive biomarkers have been identified up to now, such as tumor mutational burden (TMB), mismatch repair deficiency (dMMR), immune cell infiltration, and inhibitory receptors related gene expression such as PD-1, PD-L1, CTLA-4, LAG-3, TIM3, and TIGIT (22, 23).

**Figure 7:**
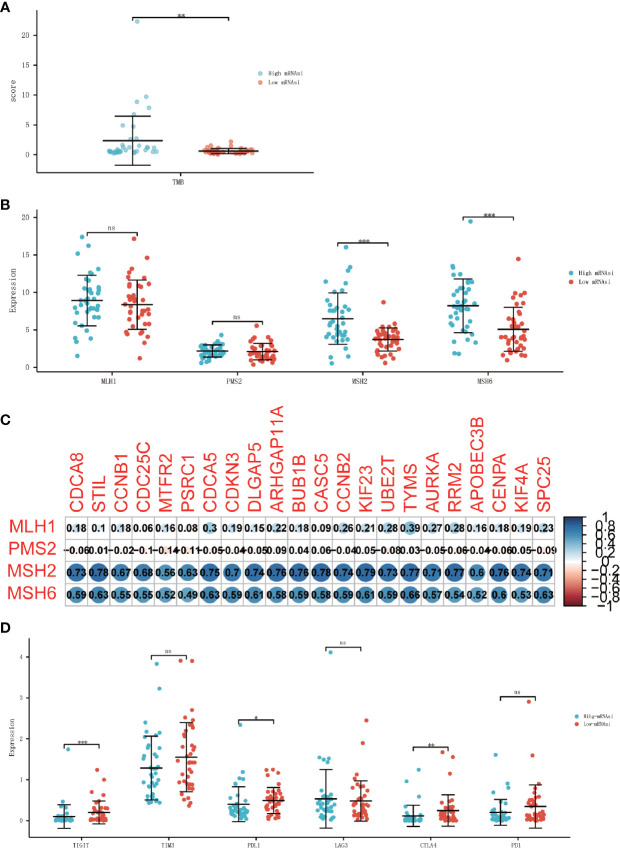
Association between ICIs biomarkers and cancer stemness. **(A)** Differential analysis of the TMB between high-mRNAsi and low-mRNAsi groups. **(B)** Differential analysis of the expression of MMR-related genes between high-mRNAsi and low-mRNAsi groups. **(C)** Correlation analysis between MMR-related genes and 22 stemness-related hub genes. **(D)** Differential analysis of the expression of immune checkpoints between high-mRNAsi and low-mRNAsi groups (PD-1, PD-L1, CTLA-4, LAG-3, TIM3, and TIGIT) (*P < 0.05, **P < 0.01, ***P < 0.001, ns, not significant).

TMB was calculated by R package “maftools”, results showed that TMB was significantly higher in the high-mRNAsi group than in the low-mRANsi group (**Figure 7A**). To determine MMR status, the expression level of four mismatch repair genes, PMS2, MSH6, MLH1, and MSH2 were analyzed, where MSH2 and MSH6 showed a significant positive correlation with the expression level of stemness-related hub genes (**Figure 7C**). The result also showed that the expression level of two mismatch repair genes (MSH2 and MSH6) in the high-mRNAsi group had a higher trend than in the low-mRNAsi group (**Figure 7B**). Since PD-1, PD-L1, CTLA-4, LAG-3, TIM3, and TIGIT had been approved as biomarkers for ICIs (24, 25), we analyzed the different expression level of them between the high-mRNAsi group and low-mRNAsi group, in which the expression of TIGHT, PD-L1, and CTLA4 decreased in the high-mRNAsi group (**Figure 7D**), indicating that ACC patients with low miRNAsi may benefit from the therapy of immune checkpoint inhibitors.

## Discussion

Cancer is increasingly a global problem, and advanced adrenocortical carcinoma is always associated with poor prognosis and high risk of recurrence (26). Surgery is the only way to cure an advanced ACC patient, therefore, to improve the prognosis of ACC patients, new effective therapeutic options are urgently needed. And improving the understanding of the key factors that promote tumor development may be important. In this study, we successfully prove that cancer stemness is significantly associated with prognosis, clinical characteristics, immune cells infiltration, and the biomarkers of ICIs. Based on the results of the above analyses, we suggest that cancer stemness could be used as a biomarker for prognosis prediction and chosen as a treatment approach for ACC patients.

Some previous research has reported that there are strong correlations between cancer stemness and cancer metastasis, drug resistance, recurrence, and poor prognosis (27–29). Our study performed comprehensive analyses of cancer stemness in ACC patients. Two parameters, mRNAsi and mDNAsi, derived from a machine learning algorithm were used to better describe the characteristics of cancer stemness. We found that high-mRNAsi was associated with poor prognosis in ACC, whereas mDNAsi showed no significant correlation with prognosis. Moreover, analyses of the relationship between clinical characteristics and mRNAsi showed a higher trend in N1 stage and metastatic tumors. The above result confirms our hypothesis that cancer stemness could be a biomarker to predict prognosis in ACC.

Tumors have strong heterogeneity and complex components. And recently, the research of cancer stem cells has sprung up. Previous research shows that cancer stem cells have similar properties to stem/progenitor cella, including self-renewal and multipotent differentiation (30). There must be some stemness-related genes that play vital roles. However, the research of therapeutic strategies targeting stemness-related genes is still in a chaotic stage and has not been developed comprehensively. Therefore, it is necessary to identify the stemness-related hub genes that can be used as therapeutic targets. Significant modules that are associated with cancer stemness and stemness-related hub genes were identified by WGCNA based on DEGs analysis between high-mRNAsi and low-mRNAsi groups. There was a significant positive correlation between the blue module and mRNAsi and a negative correlation between the blue module and ImmuneScore. By the values of MM and GS, 22 stemness-related genes were identified and proved to be overexpressed in the high-mRNAsi group. All these stemness-related genes had a strong connection at both transcription and protein levels, which indicated that the stemness-related hub genes may have strong biological links in the functions. And further GO and KEGG analyses revealed that the stemness-related hub genes were associated with cell cycle and cell mitosis, indicating that they may have the capacity of self-renewal and proliferative properties of cancer stem cells. In summary, the stemness-related genes had a high correlation with mRNAsi and ImmuneScore, which may mean they can become new therapeutic targets for the treatment of ACC patients. However, the IC50 of ACC first-line chemotherapy drugs, cisplatin and etoposide, did not differ between the mRNAsi or the expression level of stemness-related genes, which does not definitively mean that the biomarkers were not related to chemotherapy effect. Fewer samples may be one reason, further study should be preformed.

ACC is subdivided into a “lymphocyte depleted” tumor, which represents a more prominent macrophage signature, with Th1 suppressed and a high M2 response (31). Extensive evidence shows that cancer stemness was associated with reduced immune cell infiltration (32, 33). That is consistent with our findings. Deeper exploration of the causality between cancer stemness and immune cell infiltration may be helpful in the development of effective immunotherapies. We found that mRNAsi was significantly negatively correlated with the immune and stromal scores. Furthermore, the results of ssGSEA indicated that some kinds of immune cells, such as activated B cells, activated CD4 T cells, activated CD8 T cells, type 2 T helper cells, and type 17 T helper cells were correlated with the expression levels of stemness-related hub genes, whereas CD8 + T cells were negatively related. Similarly, CD8 + T cells decreased in the high-mRNAsi groups, which means that tumors may show more resistance to immunotherapy (34). The development of cancer has many influential factors, among which patients’ immunocompetence plays a crucial role, and it has been proved (35, 36). The immune system can change the behaviors of tumors through the action of cancer stem cells, as such immunotherapy targeting cancer stem cells by CD8 + T cells has been reported. Doctors can produce CSCs-specific CD8 + T cells *in vitro*, and then transfer them to patients to meet the purpose of treatment, targeting CSCs and killing the tumor cells *in vivo* (37, 38). The result that CD8 + T cell infiltration decreased in the high-mRNAsi group suggested that immunotherapy is more suitable for ACC patients with low-mRNAsi.

Immune checkpoint inhibitors have been shown to be effective for many tumors, including metastatic and localized melanoma (39, 40), and metastatic and locally advanced non-small cell lung cancer (NSCLC) (41, 42). Many efforts have been made for ACC immunotherapy (43, 44). But there is still a lack of reliable biomarkers to distinguish patients with potential sensitivity to immunotherapy. The response to ICIs is influenced by several factors, which can be monitored using various biomarkers, including degree of CD8 + T cell infiltration, tumor mutation burden, mismatch repair, and the expression of critical immune checkpoints (PD-1, PD-L1, CTLA-4, LAG-3, TIM3, and TIGIT). TMB and MMR are both major predictive biomarkers for immune checkpoint inhibitor responses, and have been proved (45, 46). One of the main challenges in using immunotherapy in ACC is the concomitant Cushing’s syndrome that occurs in 50% of patients with ACC. Glucocorticoid excess causes T cell depletion and is associated with the prognosis. And previous studies have shown that PD-L1 expression in ACC is low. It is known that correlation between tumor PD-L1 expression and response to immunotherapy has been provided for various cancer types Then, just like the results we obtained, molecular alterations that lead to an altered production of CD8 + infiltrate thus impairing the local antitumor immune response were described in ACC. Our results indicate that the patients in low-mRNAsi groups have higher PD-L1, CTLA4, and TIGHT expression, which means the patients with low-mRNAsi are more likely to benefit from immune checkpoint inhibitors.

In summary, our study reveals the comprehensive characteristics of stemness and identifies the prognosis and therapy value of mRNAsi in ACC patients. We also built a 22 stemness-related genes signature of which we checked the biological function. Analysis of the interaction between the 22 stemness-related genes and mRNAsi and immune cell infiltration may be helpful in predicting the efficacy of immunotherapy. Further analysis of PD-L1, CTLA4, and TIGHT expression contributes to the prediction of immunotherapy effectiveness. However, there are some limitations in our study. First, there are only 78 ACC samples, which may cause bias, so multicenter studies should be supplemented. Second, the conclusions are derived from retrospective data and can only result in information of correlation, not definite causation. Therefore, further investigations should focus on basic experiments and clinical trials to confirm our findings.

## Data Availability Statement

The original contributions presented in the study are included in the article/**Supplementary Material**. Further inquiries can be directed to the corresponding author.

## Author Contributions

XS and YL designed the study, HH and JZ reviewed and revised the experimental design, SC collected data and materials, and YL performed the data analysis. XS wrote the manuscript. MW and LZ provided the essential reagents and tools. SX revised the manuscript. All authors contributed to the article and approved the submitted version.

## Funding

This research was supported by the National Natural Science Foundation of China (81770488).

## Conflict of Interest

The authors declare that the research was conducted in the absence of any commercial or financial relationships that could be construed as a potential conflict of interest.

## Supplementary Material

Supplementary Figure 1 Kaplan–Meier OS curves for 22 hub genes with low and high expression based on the median cutoff point in ACC patients.

Supplementary Figure 2 Kaplan–Meier OS curves for 22 hub genes with low and high expression based on the median cutoff point in ACC patients.

Supplementary Table 1 Wilcoxon rank sum test for the Age, Gender, N stage and M stage, and Dunn’s test for the Histological type and T stage.

Supplementary Table 2 The information about clinical features.

Supplementary Table 3 Multivariate cox analysis of mRNAsi and other clinical characteristics.
