# Supplementary figures and images for: Cancer Stemness Associated With Prognosis and the Efficacy of Immunotherapy in Adrenocortical Carcinoma
Source: Front Oncol. 2021 Jul 21;11:651622. doi: 10.3389/fonc.2021.651622 (PMC8334864; doi:10.3389/fonc.2021.651622)

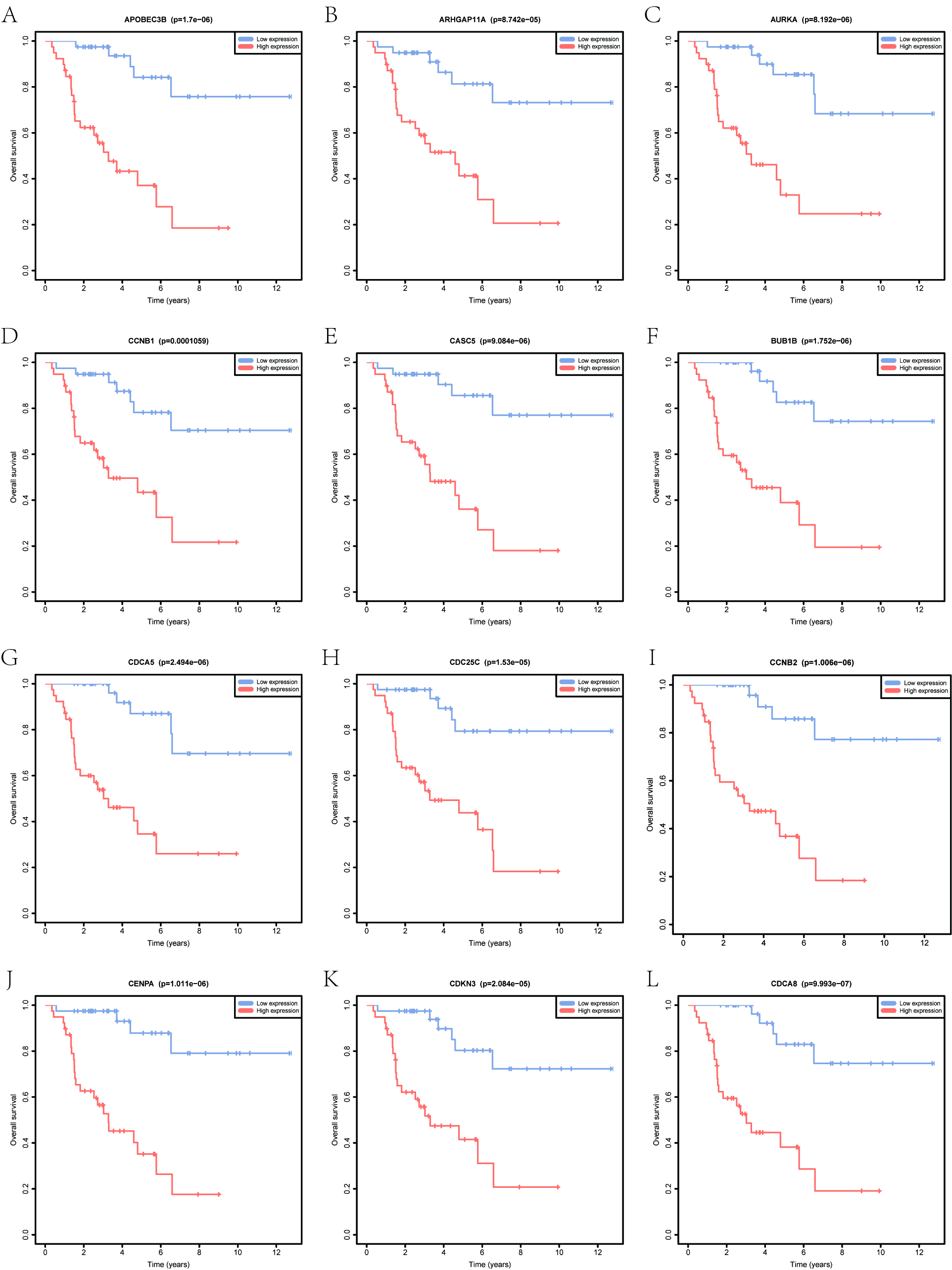

Supplement: Supplementary Figure 1 — Kaplan–Meier OS curves for 22 hub genes with low and high expression based on the median cutoff point in ACC patients. [file Image_1.tif]

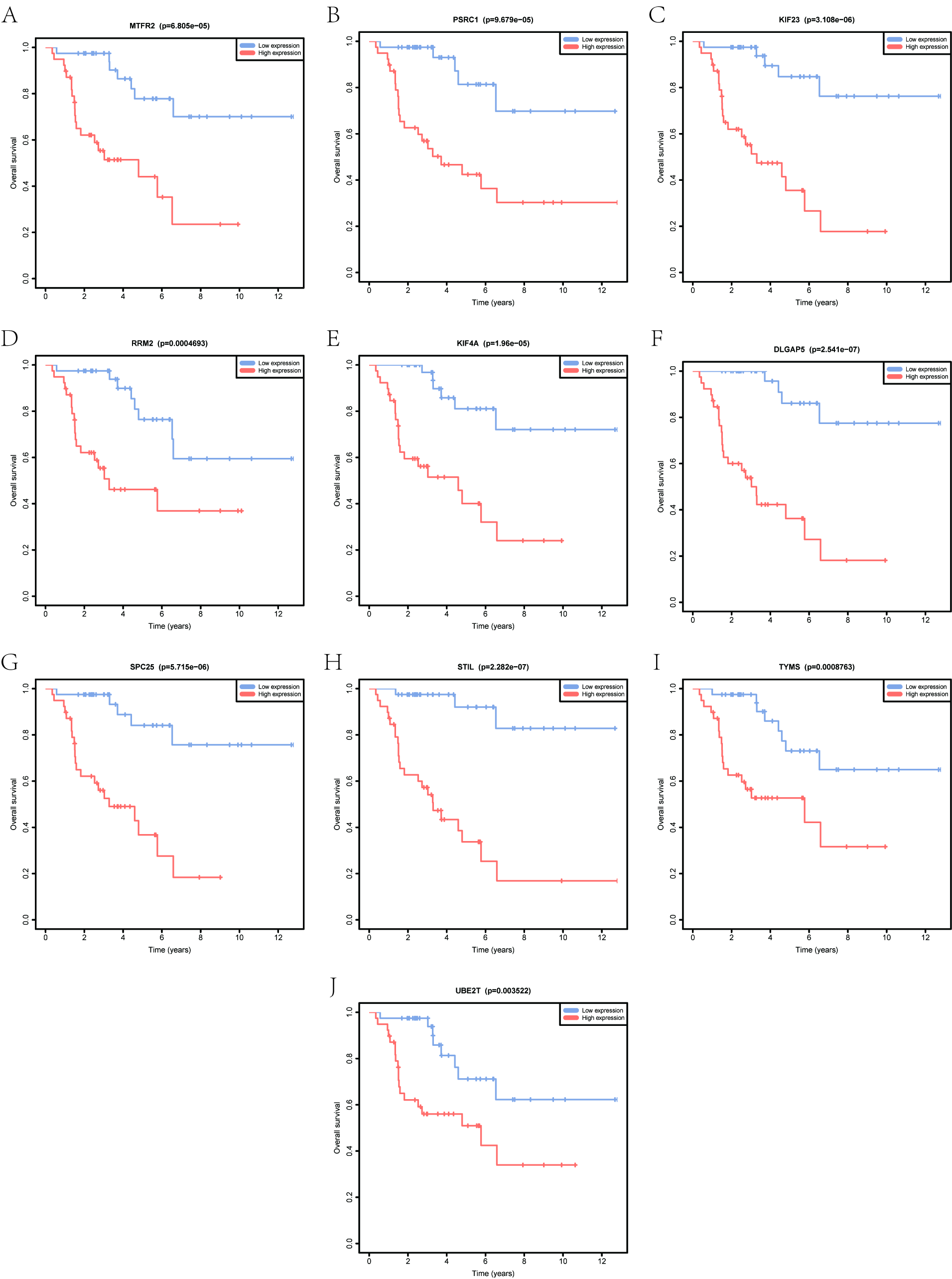

Supplement: Supplementary Figure 2 — Kaplan–Meier OS curves for 22 hub genes with low and high expression based on the median cutoff point in ACC patients. [file Image_2.tif]

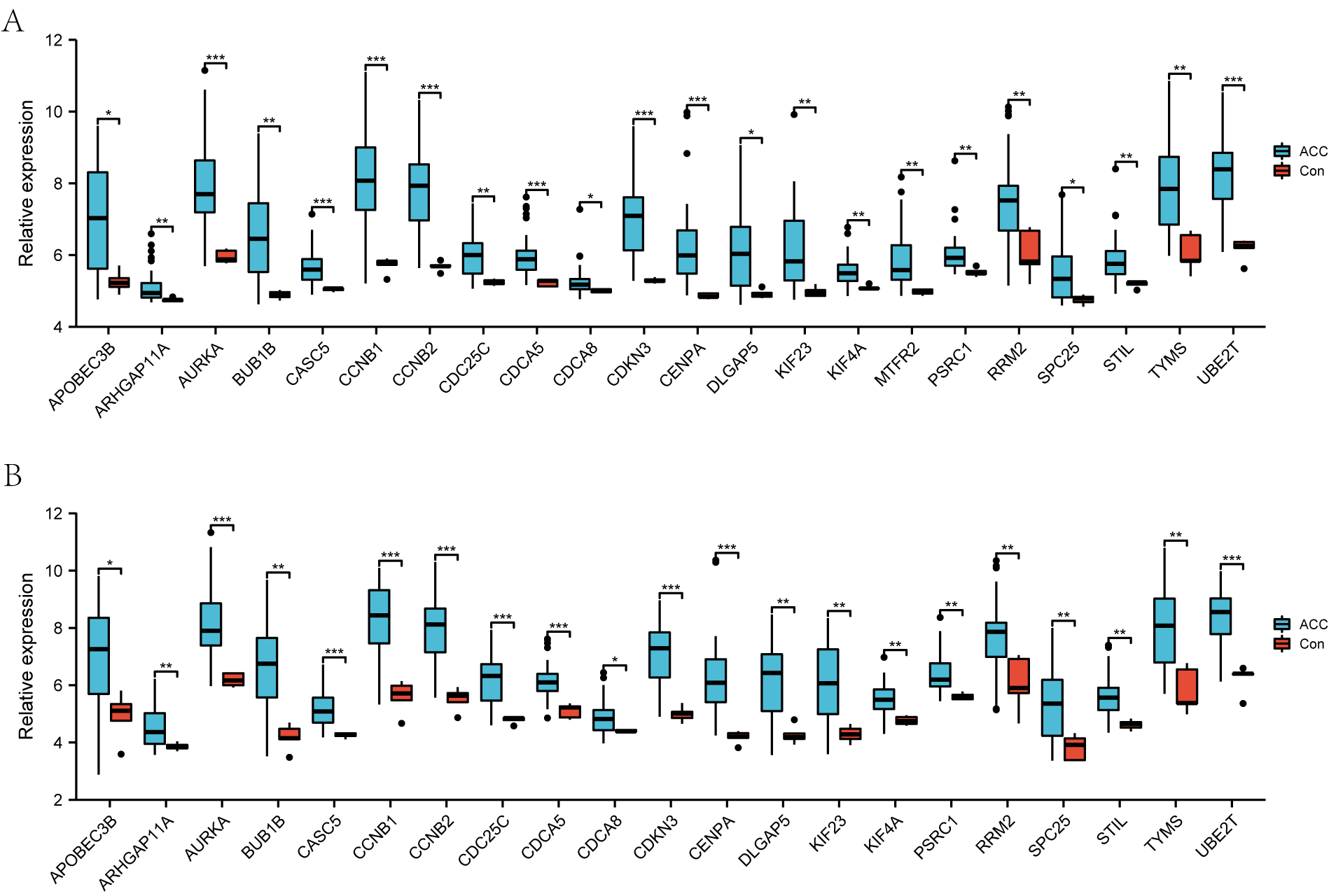

Supplement: Supplementary file 6 [file Image_3.tif]

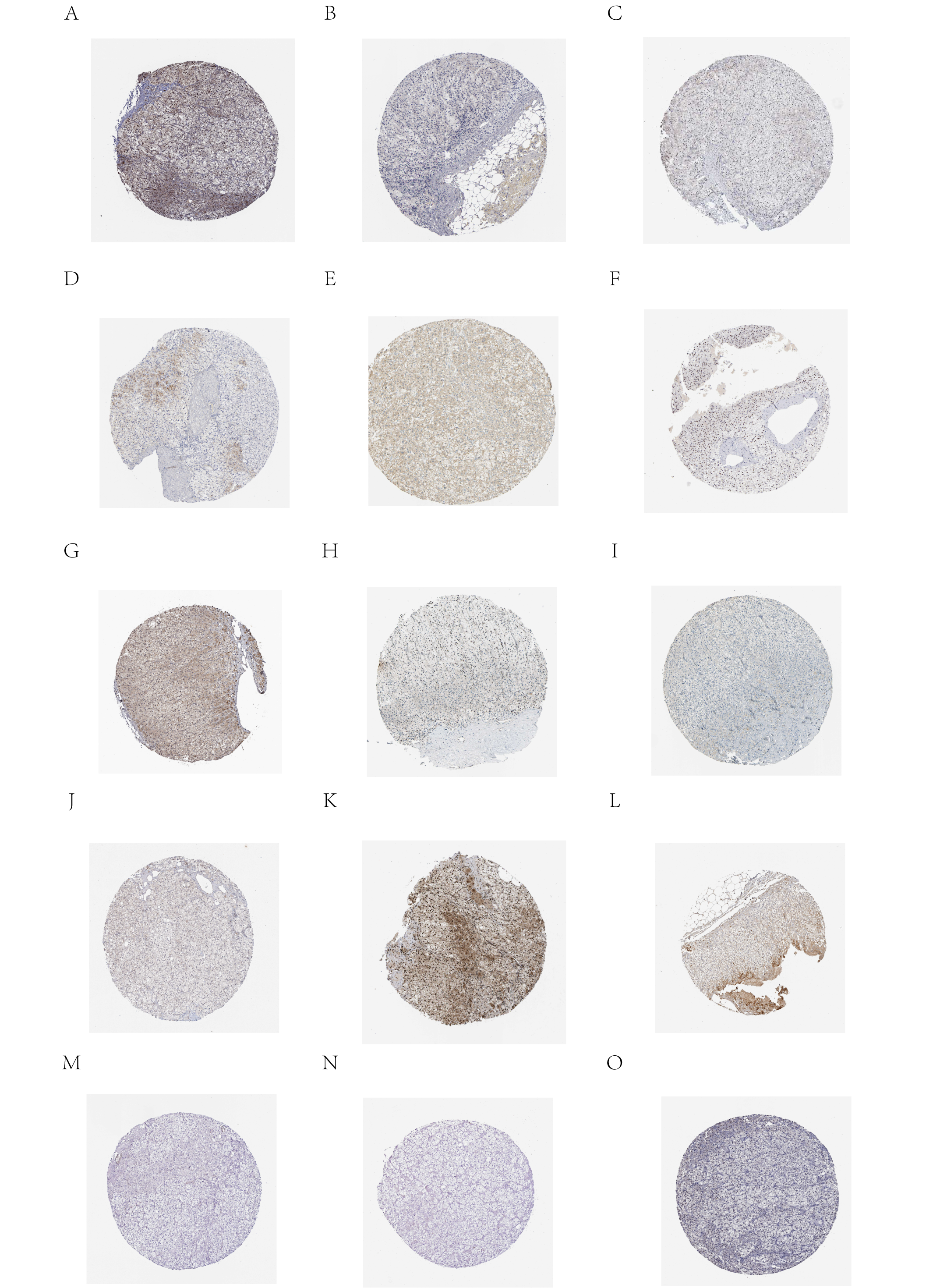

Supplement: Supplementary file 7 [file Image_4.tif]
